# DISCRIMINATION, STRESS, AND AGING ACROSS THE LIFECOURSE

**DOI:** 10.1093/geroni/igae098.1305

**Published:** 2024-12-31

**Authors:** Roland Thorpe, Carl V Hill

**Affiliations:** Chair; Discussant

## Abstract

Stress and discrimination are posited as two explanations for understanding why race disparities in health and aging across the life course remain elusive. This symposium contains papers seeking to address the impact of discrimination or stress on Black health or health disparities across the life course. First, Thierry and colleagues examined how lifetime exposure to structural stressors and current perceived neighborhood stressors are associated with cognitive function in Black adults in the HRS. They found that life course exposure to multiple domains of neighborhood-based stressors may be important for cognitive function in later life. Second, Cobb and colleagues hypothesized that self-reported instances of everyday discrimination are related to higher levels of cardiometabolic risk among older Black and White adults. The relationship between self-reported instances of everyday discrimination and cardiometabolic risk was significantly weaker for older Black adults than older White adults. Third, Mitchell and colleagues investigated how exposure to chronic stressors during mid-life indirectly affects cognitive function through changes in physiological dysregulation in HRS. Adjusting for age, gender, race/ethnicity and education fully accounted for the significant effects of financial strain and discrimination on changes in cognition. Fourth, Forrester and colleagues examined racial/ethnic differences in the association between self-reported discrimination and brain imaging in the MESA study. Findings suggest that instances of racial discrimination over a lifetime may be detrimental to brain health for Black adults. This collection of papers provides insights into how discrimination or stress impacts Black health or health disparities in middle to late life.

